# Hyponatremia as a Marker of Complicated Appendicitis: A Retrospective Analysis

**DOI:** 10.7759/cureus.26672

**Published:** 2022-07-08

**Authors:** Jonathon Sheen, Joel Bowen, Helen Whitmore, Kirk Bowling

**Affiliations:** 1 General Surgery, Torbay and South Devon NHS Foundation Trust, Torquay, GBR; 2 Upper GI Surgery, Torbay and South Devon NHS Foundation Trust, Torquay, GBR

**Keywords:** complicated appendicitis, diagnostic tool, appendicectomy, hyponatraemia, appendicitis

## Abstract

Background

The aim of this study is to investigate the potential role of hyponatremia as a biochemical predictor of complicated appendicitis. The effective employment of biochemical markers to identify early and predict progression to complicated appendicitis would be beneficial in triaging those most requiring urgent appendicectomy. A marker of interest and subject of recent study in the literature is sodium.

Methods and Materials

This study was designed as a single-center, retrospective analysis of all appendicectomies performed between January 1, 2018 and March 10, 2021. Patients were categorized into pediatric and adult groups and subdivided into uncomplicated or complicated appendicitis. We utilized the Chi-square test and crude odds ratio (OR) rates to assess significance of serum sodium level values.

Results

In total, 890 patients underwent appendicectomy (181 pediatric, 709 adult cases). Within the pediatric group, 10 uncomplicated cases and 16 complicated cases were found to be hyponatremic. The result for hyponatremia as a diagnostic marker for complicated cases in this group was not significant at p<0.05, with a Chi-square test result of 1.6067 and p-value 0.204963 (OR 1.7538, 95% confidence interval (CI) 0.7312-4.2070). Adults displaying hyponatremia comprised four uncomplicated and 34 complicated cases, with calculated OR 7.915 (95% CI 2.7656-22.6521). Chi-square test result was 20.1687 with a p-value of <0.00001 and, thus, statistically significant.

Conclusion

Our findings suggest that hyponatremia can be employed as an indicator of complicated appendicitis in an adult population. This correlates with the findings of a recent systematic review of this topic and implicates this as a subject worthy of further study.

## Introduction

Acute appendicitis is the most common general surgical emergency in the world, with 50,000 appendicectomies performed in the UK per annum [1,2]. Whilst it can affect individuals of any age, acute appendicitis is more common in males aged 10 to 20 years. Interestingly, however, women are twice as likely to undergo appendicectomy [1,3,4]. The lifetime risk of developing the condition is 8.6% in males and 6.9% in females [1,3].

Despite the high prevalence of the condition, the definitive pathophysiology leading to acute appendicitis remains subject to debate and is not completely understood [5,6]. The severity of the condition can vary considerably, from subclinical and self-resolving, to fulminant sepsis and death. This variability in presentation makes it pertinent to categorize cases as either 'uncomplicated' or 'complicated' [7,8]. Exact methods of categorization vary and definitions can be inconsistent across the literature. However, for the purpose of this paper, 'uncomplicated' is to include simple, focal, or suppurative cases, whereas ‘complicated’ describes a gangrenous or perforated appendix, as well as those displaying peritonitis or periappendiceal abscess formation [7,8]. The extent of pathology has a marked, measurable impact on a patient’s outcome: the mortality rate where perforation has occurred remains around 5%, compared to 0.1% in 'acute but not gangrenous' appendicitis [5,9]. The rate of perforation has been reported at between 16-40%, with a significantly greater risk at the extremes of age [1,9].

A combination of clinical observation, measurement of inflammatory markers, and imaging modalities are typically required to both diagnose and prognosticate the presence of and severity of acute appendicitis. Multiple scoring systems have been devised to include these, and whilst they have been validated to aid risk stratification in acute appendicitis, none has been widely accepted into clinical practice [9-11].

Given the prevalence of the condition and significant morbidity and mortality associated with appendicitis, the effective employment of biochemical markers to allow early identification and predict progression to complicated appendicitis would likely carry significant benefit to many. An increased index of suspicion could potentially aid in correctly triaging those who would benefit most from urgent appendicectomy. A marker of interest and subject of recent study in the literature is sodium. Attractive due to its accessibility, low cost, and inclusion in baseline biochemical measurements in most secondary care settings, an association between hyponatremia and complicated appendicitis has been reported. Of particular note, a recent systematic review of seven existing papers exploring the link between hyponatremia and appendicitis, four papers demonstrated a significant association between low serum sodium and complicated appendicitis in both pediatric and adult populations [12].

The aim of this study is to further investigate the potential role of hyponatemia as a biochemical predictor of complicated appendicitis.

## Materials and methods

Study design

This study was designed as a single-center, retrospective analysis of all appendicectomies performed between January 1, 2018 and 10 March, 2021.

Patient data was collected through our operating theater database, and collated into a single spreadsheet. The initial data set included all patients treated operatively with either open or laparoscopic appendicectomy. For each patient included in the study, admission documentation, operation note, discharge summary, blood results and histology findings were reviewed.

Of note, the blood tests analyzed were the admission blood tests for each patient. Our patients presented to either our emergency department or our surgical receiving unit. In each of these two areas, the blood tests are taken immediately as part of the initial assessment.

Patients were then categorized into pediatric (17 years and younger) and adult (18 years and above) groups, and then subdivided into either uncomplicated or complicated appendicitis. As outlined in the Introduction, the accepted definition of ‘uncomplicated’ was histological confirmation of simple, focal or suppurative appendicitis. The ‘complicated’ group comprised cases confirmed histologically or radiologically as gangrenous or perforated appendicitis, or any showing peritonitis or periappendiceal abscess formation.

For the purpose of this study, we defined hyponatremia as a serum sodium level of less than 135 mmol/L, for both adult and pediatric populations, in keeping with the guidelines of our center and with the UK's National Institute for Clinical Excellence (NICE) guidance [13]. For pro-inflammatory markers, we defined raised C-Reactive Protein (CRP) as more than 5 mg/L, raised White Cell Count (WCC) as more than 10 x 10^9^/L and raised neutrophils as more than 7.5 x 10^9^/L, as per guidelines at our center.

Exclusion criteria

Patients were excluded from statistical analysis if appendicectomy was performed as an elective procedure (either interval appendicectomy or as part of another elective procedure: for example, hysterectomy). Other exclusion criteria applied were: if the appendix was removed during an emergency procedure performed to treat another pathology, if the histology sample was missing, or if notes were incomplete. Finally, if histological findings confirmed a pathology other than appendicitis (e.g., macroscopically normal, tumor, helminth, fibrosis), the case was also excluded from analysis.

Statistical analysis

Data was collated into a single, formatted spreadsheet on Microsoft Excel 2019. We assessed the significance of serum sodium level values by using the Chi-square test and crude odds ratio (OR) rates, utilizing tools on soscistatistics.com.

We then performed further analysis of the pro-inflammatory markers on the hyponatremic patients in both the adult and pediatric cohorts. This analysis was performed to assess for any statistical significance, to value of p<0.05, between the complicated and uncomplicated groups for raised CRP, raised WCC, and raised neutrophil count; for this we used Fisher’s exact test, again using tools on soscistatistics.com.

## Results

Between January 1, 2018 and March 10, 2021, a total of 890 patients underwent appendicectomy at our center. Of these, 181 were pediatric cases and 709 were adult cases.

On initial analysis, 46 pediatric cases were excluded: 37 macroscopically normal, one elective appendicectomy, two tumors, four helminths, one serosal inflammation only, and one no histological sample. 194 adult cases were excluded: 136 macroscopically normal, 20 elective cases, 17 tumors, two helminths, 14 chronic inflammation/fibrosis/serosal inflammation only, three no histological sample, one incomplete notation, and one diagnostic laparoscopy only.

Of the remaining 135 paediatric cases, 67 were uncomplicated appendicitis and 68 complicated (Table 1).

**Table 1:** Paediatric case data

Paediatric	Uncomplicated	Complicated
Total	67	68
Mean age (years)	12.91	13
Age range (years)	6-16	6-17
Mean sodium (mmol/L)	137.84	136.53
Sodium range (mmol/L)	130-42	129-43
Hyponatremic	10	16
Mean hyponatremia (mmol/L)	133.40	132.06
Mean age (years)	11.3	12.5

10 uncomplicated cases and 16 complicated cases were found to be hyponatremic, with the respective means being 133.40mmol/L and 132.06mmol/L. A calculated OR between the two groups is 1.7538 (95% confidence interval (CI) 0.7312-4.2070), meaning p=0.2082. The Chi-square test statistic result is 1.6067, with a p-value of 0.204963. Therefore, the result for hyponatremia as a diagnostic marker for complicated appendicitis cases in the pediatric cohort at our center is not significant as p>0.05.

Of the remaining 515 adult cases, 234 were uncomplicated and 281 complicated (Table 2).

**Table 2:** Adult case data

Adult	Uncomplicated	Complicated
Total	234	281
Mean age (years)	41.913	46.591
Age range (years)	18-83	18-90
Mean sodium (mmol/L)	139.11	138.02
Sodium range (mmol/L)	127-45	108-48
Hyponatremic	4	34
Mean hyponatremia (mmol/L)	132.00	131.29
Mean age (years)	54	54.382

On analysis of these cases, four uncomplicated cases and 34 complicated cases were found to be hyponatremic, with the respective means being 132.00mmol/L and 131.29mmol/L. A calculated OR between the two groups is 7.915 (95% CI 2.7656-22.6521), meaning p=0.0001. The Chi-square test statistic result is 20.1687, with a p-value of <0.00001. Therefore, the result for hyponatremia as a diagnostic marker for complicated appendicitis cases in the adult cohort at our center is significant at p<0.05 (Table 3).

**Table 3:** Summary of statistical analysis for hyponatremia OR: Odds Ratio

Cohort	OR	Chi-square	Significant at p<0.05?
Pediatric	1.7538 (95% CI 0.7312-4.2070); p=0.2082	1.6067; p=0.204963	No
Adult	7.915 (95% CI 2.7656-22.6521); p=0.0001	20.1687; p<0.00001	Yes

Following this initial analysis, we looked further into the pro-inflammatory markers for patients with hyponatremia. We analyzed each of these patients for raised CRP (>5 mg/L), raised WCC (>10 x 10^9^/L), and raised neutrophils (>7.5 x 10^9^/L).

Looking firstly at the pediatric cohort, 10 uncomplicated cases and 16 complicated cases were found to be hyponatremic. Of the 10 uncomplicated cases, all were found to have a raised CRP, nine were found to have a raised WCC, and nine were found to have a raised neutrophil count. For the 16 complicated cases, all 16 had a raised CRP, 15 had a raised WCC, and 15 had a raised neutrophil count. We analyzed each of CRP, WCC, and neutrophil counts using Fisher’s exact test. None of them were found to be statistically significant (Table 4).

**Table 4:** Pediatric cohort inflammatory marker analysis CRP: C-Reactive Protein; WCC: White Cell Count

Pediatric	Fisher's exact test	Significant at p<0.05?
Raised CRP	1	No
Raised WCC	1	No
Raised neutrophil count	1	No

In the adult cohort, four uncomplicated cases and 34 complicated cases were found to be hyponatremic. Of the four uncomplicated cases, three were found to have a raised CRP, two were found to have a raised WCC, and two were found to have a raised neutrophil count. For the 34 complicated cases, 33 had a raised CRP, 27 had a raised WCC, and 27 had a raised neutrophil count. As per the pediatric cohort, we analyzed each of CRP, WCC, and neutrophil count using Fisher’s exact test. None of these showed a statistically significant difference between the complicated and uncomplicated groups (Table 5).

**Table 5:** Adult cohort inflammatory marker analysis CRP: C-Reactive Protein; WCC: White Cell Count

Adult	Fisher's exact test	Significant at p<0.05?
Raised CRP	0.202	No
Raised WCC	0.232	No
Raised neutrophil count	0.232	No

## Discussion

Appendicitis is a common condition, which can be difficult to diagnose. More complicated pathology presents potential serious harm to the patient, so additional markers to assist in the diagnosis would present a useful aid. A systematic review of previous studies investigating this topic found some evidence of a link between serum sodium level and complicated pathology [12]. Our findings suggest that at our center, hyponatremia is a greater significant predictive factor than inflammatory markers (CRP, WCC, and neutrophil count) for complicated appendicitis in adults but not in our pediatric cohort.

Several predictive tools for the diagnosis of appendicitis already exist, none of which utilize serum sodium levels [9-11]. These existing tools integrate levels of inflammatory components of blood results alongside signs and symptoms characteristic of appendicitis. These tools are used to assess the diagnostic likelihood of appendicitis rather than looking to establish whether it could be complicated or uncomplicated appendicitis. Whilst it may be the case that more work is required to investigate the sensitivity and specificity, given the findings of our study, we feel it would be pertinent to consider the integration of hyponatremia into a diagnostic tool for cases of suspected complicated appendicitis. Such a diagnostic tool would require appropriate validation, but as we have been unable to find one that utilizes the presence or absence of hyponatremia, we believe that this could be a beneficial development, particularly with the additional pressures that global healthcare is currently under.

The exact etiology of hyponatremia in those with appendicitis is not well understood, but it has been proposed that interleukin-6 (IL-6) plays a key role [14]. Pro-inflammatory cytokines, including IL-6, are released by damaged and inflamed tissues. Circulating IL-6 is then either transported or diffuses across the blood-brain barrier. From here, it appears that IL-6 activates the subfornical organ and the organum vasculosum, resulting in thirst and also acting as a non-osmotic stimulus for release of antidiuretic hormone (ADH) from the supraoptic nucleus and paraventricular nucleus of the hypothalamus. ADH then acts upon the distal convoluted tubule and the collecting duct of nephrons within the kidney by inserting aquaporins. These aquaporins increase free water reabsorption, leading to a dilutional hyponatremia [14].

Due to this potentially confounding link between inflammation and hyponatremia, we felt it pertinent to look at the pro-inflammatory markers in the patients we found to be hyponatremic. Our data showed that the majority of patients in both the uncomplicated and complicated groups of both cohorts had raised CRP, raised WCC, and raised neutrophil counts. However, further analysis showed no significant difference between the hyponatremic complicated and uncomplicated groups. These results suggest that whilst raised inflammatory markers are a good predictor in the toolbox for appendicitis, hyponatremia may play a greater role in distinguishing between complicated and uncomplicated appendicitis.

As mentioned above, our study showed significant findings in the adult population at our center, but did not in the pediatric group. A search of the literature shows that previous papers investigating the link between hyponatremia and complicated appendicitis have concentrated on either an adult population or a pediatric population, so this phenomenon has not been encountered in previous studies [15-17]. We propose a number of explanations for this. The total case number in our pediatric population is 135, compared to 515 in our adult population. It could be that a larger pediatric population would have resulted in a significant result. An adult population is also more likely to have other risk factors for hyponatremia, such as comorbidities and medications, which could result in an increase in the impact of complicated appendicitis on serum sodium levels [18]. It may also be the case that pediatric patients maintain homeostasis, despite the physiological insult of complicated appendicitis, for longer than their adult counterparts. From a first principles scientific perspective, this seems logical, as the physiological reserve of a child is well recognized to exceed that of the frail and comorbid adult patient.

In addition to the relatively low number of pediatric cases, our study has a number of other limitations. These being that our data was collected retrospectively, our data only includes those undergoing appendicectomy, and the potential confounding factor of cases occurring during the COVID-19 pandemic. The impact of the pandemic has likely been extensive and multifactorial. However, one important limitation that must be considered as a potential source of bias in our results is that in early 2020, as the first wave of COVID-19 struck, our center, along with many others, placed a far greater emphasis on the use of non-operative management of appendicitis with intravenous antibiotics, particularly in uncomplicated cases. This was an uncommon strategy in UK healthcare prior to the pandemic, but due to concerns about the transmissibility of the virus during laparoscopic procedures, advice was issued by the Royal College of Surgeons of England and the Association of Upper Gastrointestinal Surgery of Great Britain and Ireland to employ non-operative management in all but perforated cases, which were typically managed via an open approach [19,20]. As a result, our number of uncomplicated cases during this period may be artificially low, as our analysis was of those undergoing operative management. However, we believe that our large sample size will minimize the impact of this possible skew.

## Conclusions

This study was conducted with the intention of assessing the role of serum sodium level as a predictor and indicator of progression to complicated appendicitis. We have found a statistically significant result in the adult population and, based on this, would recommend utilizing it as another item in the diagnostic toolbox. Alongside careful clinical assessment, measurement of inflammatory markers, and appropriate use of imaging, we believe that assessment of a patient’s sodium level can provide an extra piece to the puzzle.

As an element of routine baseline blood testing in most centers, there is no extra cost involved, and so without an additional outlay, correct highlighting of hyponatremia in suspected appendicitis could provide a crucial early indication of a patient at risk of complication. Our findings, in keeping with the preceding literature, show promise for this potential biochemical marker and further study is warranted.
